# Effect of CYP3A4 and PPARA polymorphism on concentration-to-dose ratio and adverse effects of tacrolimus in Pakistani liver transplant recipients

**DOI:** 10.12669/pjms.38.7.6180

**Published:** 2022

**Authors:** Fahad Azam, Moosa Khan, Abida Shaheen, AbuBakar Hafeez Bhatti

**Affiliations:** 1 Fahad Azam, MBBS, M.Phil., PhD Associate Professor, Pharmacology & Therapeutics, Shifa Tameer-e-Millat University, Islamabad, Pakistan; 2 Moosa Khan, MBBS, M.Phil., PhD Professor, Pharmacology & Therapeutics Shaheed Zulfiqar Ali Bhutto Medical University, Islamabad, Pakistan; 3 Abida Shaheen, MBBS, M.Phil., PhD Professor, Pharmacology &Therapeutics, Shifa Tameer-e-Millat University, Islamabad, Pakistan; 4 Abu Bakar Hafeez Bhatti, FCPS, FRCS. Consultant, Liver Transplant, Shifa International Hospital, Islamabad, Pakistan

**Keywords:** Tacrolimus, liver transplant, adverse effects, immunosuppression

## Abstract

**Objectives:**

Polymorphism in cytochrome P450 3A4 (CYP3A4) and its regulatory gene peroxisome proliferator activated receptor (PPARA) may significantly affect the metabolism of tacrolimus. This study aims to explore the effect of the single nucleotide polymorphisms (SNP) of cytochrome P450 3A4 and PPARA on the pharmacological variables of adverse effects and the concentration-to-dose ratio (CDR) of the immune suppressant drug tacrolimus in Pakistani liver transplant recipients.

**Methods:**

Eighty-one liver transplant patients were included and their demographic and clinical data were recorded. Dosages and trough levels of tacrolimus measured by electrochemiluminescence (ECLIA) were recorded daily. Genotyping for transplant recipients was performed for CYP3A4 rs35599367, PPARA rs4253728 and rs4823613. Incidence of sepsis, acute cellular rejection (ACR) and other adverse effects were recorded.

**Results:**

Liver transplant recipients with CYP3A4 rs35599367 CT and TT genotype reported higher tacrolimus CDR compared to the CC genotype during week-1 (p<0.001) and week-2 (*p* =0.03) post-transplantation period. CYP3A4 rs35599367 polymorphism presented a significant association with nephrotoxicity, sepsis, seizures and psychosis. Significant association of PPARA rs4253728 and PPAARA rs4823613 polymorphism with ACR was observed.

**Conclusion:**

Genotyping for CYP3A4 rs35599367 polymorphism during dose titration may shorten the duration to reach optimal tacrolimus trough levels and may help predict adverse events in transplant recipients receiving tacrolimus; genotyping for PPARA rs4253728 and rs4823613 may predict the incidence of ACR.

## INTRODUCTION

Liver transplantation (LT) is a radical life-saving practice that requires the initiation of immune suppressant regimens to improve clinical outcomes.^1^ Tacrolimus is a popular drug to suppress the immune system in liver transplant recipients by inhibiting the calcium-dependent phosphatase enzyme calcineurin that results in the blocking of signal-2 consequently blocking the T-cell activation.^2^

The use of tacrolimus requires continuous monitoring of trough levels followed by frequent dose titration as it has a narrow therapeutic window and is subject to inter-individual variability and serious adverse effects.^3^ The variable efficacy and adverse effects associated with tacrolimus despite close monitoring of blood levels and repeated dose adjustments hint towards a possible role of polymorphism in the genes associated with absorption, metabolism or excretion of this drug.^4^ Polymorphism in CYP3A4 and CYP3A5 may significantly affect the metabolism of tacrolimus thereby altering its blood levels; the role of polymorphism in the peroxisome proliferator-activated receptor-α (PPARA) gene has been reported as it regulates transcription and expression of CYP3A4 and CYP3A5 genes.^5^,^6^

Polymorphism in the CYP3A4*22 allele at rs35599367 results in reduced expression of the CYP3A4 enzyme consequently resulting in variability in the pharmacokinetics of calcineurin inhibitor drugs tacrolimus and cyclosporine.^7^ Polymorphism in the two linked SNPs rs4253728 and rs4823613 of the PPARA gene can influence the levels and function of CYP3A4 enzymes.^8^ Titration of tacrolimus doses consumes precious time and resources in the clinical setting; therefore it is important to gain insight into the effect of genetic polymorphism to administer personalized doses of tacrolimus. Currently, there is no data regarding the influence of polymorphism in CYP3A4, CYP3A5 and PPARA on tacrolimus trough levels during the initial immune suppression in Pakistani liver transplant recipients.

With this context, this study aims to evaluate the effect of polymorphism in CYP3A4 rs35599367, PPARA rs4253728 and PPARA rs4823613 on the adverse events and the dosages of tacrolimus in Pakistani liver transplant recipients.

## METHODS

The present study was conducted at Shifa Tameer-e-Millat University and Shifa International Hospital in collaboration with the Basic Medical Sciences Institute from September-2016 to June-2021 after obtaining the Institutional Review Board approval (IRB #638-086-2016). After obtaining written informed consent, eighty-one patients were included in this study; all transplantations were conducted after taking permission from the Human Organ Transplant Authority (HOTA) in accord with the Helsinki declaration. Demographic data and relevant variables were recorded at baseline and laboratory investigations were daily monitored. Details of the assessment process of the recipients and donors are described elsewhere.^9^,^10^

The immune suppression regimen was initiated 8-12 hours after the transplantation procedure using the nasogastric or oral route. As per the routine practice, tacrolimus and steroids were used as the standard regimen for the initiation of immune suppression; 0.5 mg/day was the initial dose of the tacrolimus on the first post-transplantation day.

The trough concentration of tacrolimus in 5-mL venous blood of patients in EDTA tubes was measured by Electrochemiluminescence immunoassay (ECLIA); daily adjustments in tacrolimus dosing were guided by the tacrolimus trough levels, relevant clinical indices and reporting of adverse effects. Laboratory investigations for relevant variables were daily evaluated. The ratio of the tacrolimus pre-dose concentration (C0) and the corresponding daily tacrolimus dose (D) was computed to obtain the concentration-to-dose ratio (CDR).^11^

The genomic-DNA was separated by the phenol and proteinase-K method. Following sequences were used to develop primers: PPARA(42) rs4253728, (forward 5′- TCTCCCAGTCTGTGGCTTGT-3′; reverse 5′-ATCTCCGGACCCACACATC-3′); PPARA(48) rs4823613 (forward 5′-CTGACAGAGGTAAGGCTT-3′; reverse 5′- ATTTAGATGGGAAGCACA-3′); CYP3A4*22 rs35599367 (forward 5′- CTGAAGAGGAATCGGCTCTG-3′; reverse 5′-TTTTTACCATCCTTCCTCTATGC-3′).

PPARA rs4253728 augmented fragment length was 372bp and sliced with ECORI into 210 and 162bp to consequent GG, GA and AA genotype PCR products. PPARA rs4823613 with 104bp was sliced by Tsp5091 into 80 and 24 with PCR products of GG, AG and AA and CYP3A4*22 rs35599367 with a measurement of 397bp was sliced with DraIII into 244bp and 153bp with TT, CT and CC products.

Numerical data were presented as mean ± standard deviation (SD). Nominal data were described as values and were analyzed by Pearson’s χ^2^ test. Analysis of the genotype data analysis and quality control was conducted by SPSS version 24.0. A two-tailed P-value of <0.05 was considered significant.

## RESULTS

Eighty-one (twenty-one female and sixty male) liver recipients were enrolled. The mean age of the recipients was 47.82±10.02 with a mean body mass index of 26.35±4.67 kg/m2. The mean CDR of tacrolimus in week-1 was 2.43±1.10 ng/ml/mg/day. HCV (49.3%) was the most prevalent aetiology for liver transplantation followed by HBV (14.8%). The most common ethnic group was Punjabi (37%) followed by Pushto speaking population (22%). Other demographic and clinical data are provided in Table-I.

**Table I:** Clinical characteristics and Demographic data.

Variables	Recipients (n=81) (Mean±SD)
Age(years)	47.82(10.02)
Body mass index(kg/m^2)^	26.35(4.67)
MELD score	21.77(5.39)
***Tacrolimus(CDR) (ng/ml/mg/day)***	
Week-1	2.39±2.02
Week-2	3.33±2.27
Week-3	1.99±1.19
Week-4	2.01±0.90
Mean	2.43±1.10
*Demographic data*	*n (%)*
Male recipients/female recipients	60(74.0) / 21(25.9)
***Ethnicity***	
Urdu Speaking	11(13.6)
Sindhi	13(16.0)
Pathan	18(22.2)
Punjabi	30(37.0)
Others	9(11.1)
*Aetiology*	*n(%)*
HCC, Cryptogenic liver cirrhosis	2(2.46)
HDV	8(9.87)
HBV, HDV	12(14.81)
HBV, ESLD	12(14.81)
HCV	40(49.38)
Other	7(8.64)

There was a significant difference in CDR of CT and TT genotype at CYP3A4 rs35599367 compared to CC genotype during week-1 (2.8±0.8 ng/ml/mg/day and 3.5±2.2 ng/ml/mg per day vs 1.6±0.8 ng/ml/mg/day; p<0.001) and week 2 (2.4±0.6 ng/ml/mg per day and 2.3±0.8 ng/ml/mg per day vs 1.7±0.9 ng/ml/mg per day; p=0.003).

Differences in CDR of genotype GG, GA and AA individuals at PPARA*42 rs4253728 and GG, GA and AA individuals at PPARA*48 rs4823613 were not significant. Comparison of CDR of participants with different genotypes at CYP3A4*22 rs35599367, PPARA*42 rs4253728 and PPARA rs4823613 are shown in Table-II.

**Table II:** Post-transplantation Tacrolimus-CDR for each genotype.

RS-numbers	Week	Tacrolimus-CDR in Transplant Recipients			P-value
CYP3A4*22 rs35599367		CC (n=43)	CT (n=25)	TT (n=8)	
	Week-1	1.63±0.85	2.80±0.84	3.51±2.26	.001*
	Week-2	1.73±0.9	2.42±0.61	2.39±0.82	.003*
	Week-3	1.87±1.07	2.53±1.74	1.71±0.34	0.098
	Week-4	2.85±2.07	4.0±2.76	3.09±1.18	0.134
	Mean	2.02±1.0	2.94±1.17	2.67±0.84	0.003*
PPARA42 rs4253728		GG(n=52)	GA(n=8)	AA(n=11)	
	Week-1	2.22±1.65	3.39±2.14	1.98±1.90	0.177
	Week-2	3.53±2.42	4.28±2.71	2.15±1.08	0.110
	Week-3	2.1±1.19	1.73±0.75	1.69±1.61	0.497
	Week-4	2.13±0.85	2.31±1.02	1.56±0.96	0.120
	Mean	2.5±1.09	2.92±1.27	1.84±0.90	0.089
PPARA48 rs4823613		AA(n=23)	GA(n=50)	GG(n=5)	
	Week-1	2.57±2.05	2.37 ± 2.04	1.62±2.06	0.641
	Week-2	3.33±2.14	3.38±2.44	2.31±0.86	0.614
	Week-3	2.15±1.28	1.93±1.15	1.67±1.56	0.663
	Week-4	2.08±0.96	2.03 ± 0.90	1.37±0.71	0.279
	Mean	2.53±1.10	2.43±1.14	1.74±0.55	0.357

* =statistically significant.

The incidence of psychosis, seizures and nephrotoxicity in CC individuals at CYP3A4*22 rs35599367 was significantly less than CT and TT recipients. GG individuals with PPARA*42 rs4253728 and PPARA*48 rs4823613 reported less acute cellular rejection (ACR) in comparison to GA and GG genotypes. Adverse effects and ACR with reference to the genotypic polymorphism is presented in Table-III.

**Table III:** Tacrolimus related adverse effects for each genotype.

Genotype	Adverse Effects n(%)		Psychosis n(%)		Seizures n(%)		Nephrotoxicity n(%)		ACR n(%)		Sepsis n(%)	
CYP3A4 rs35599367	(+)	(-)	(+)	(-)	(+)	(-)	(+)	(-)	(+)	(-)	(+)	(-)
CC(n=43)	10(13.1)	33(43.4)	7(9.2)	36(47.3)	1(1.3)	42(55.2)	1(1.3)	42(55.2)	3(3.9)	40(46.0)	8(10.5)	35(46.0)
CT(n=25)	11(14.4)	14(18.4)	9(11.8)	16(21.0)	5(6.5)	20(26.3)	11(14.4)	14(18.4)	0(-)	25(32.8)	9(11.8)	16(21.0)
TT(n=8)	3(3.9)	5(6.5)	0(-)	8(10.5)	3(3.9)	5(6.5)	0(-)	8(10.5)	0(-)	8(10.5)	6(7.8)	2(2.6)
p-value	0.19		0.05*		0.003*		< 0.001		0.495		0.005*	

*Genotype*	*Adverse Effects n(%)*		*Psychosis n(%)*		*Seizures n(%)*		*Nephrotoxicity n(%)*		*ACR n(%)*		*Sepsis n(%)*	

PPARA 42 rs4253728	(+)	(-)	(+)	(-)	(+)	(-)	(+)	(-)	(+)	(-)	(+)	(-)
AA(n=11)	6(8.4)	5(7.0)	2(2.8)	9(12.6)	2(2.8)	9(12.6)	3(4.22)	8(11.26)	3(4.2)	8(11.2)	3(4.2)	8(1.2)
GA(n=8)	2(2.8)	6(8.4)	2(2.8)	6(8.4)	2(2.8)	6(8.4)	1(1.40)	7(9.85)	1(1.4)	7(9.8)	2(2.6)	6(8.4)
GG(n=52)	18(25.3)	34(47.8)	13(18.3)	39(54.9)	5(7.0)	47(66.1)	7(9.8)	45(63.38)	7(9.8)	45(63.3)	14(19.7)	38(53.5)
p-value	0.40		1.0		0.34		0.49		0.03		1.0	

*Genotype*	*Adverse Effects n(%)*		*Psychosis n(%)*		*Seizures n(%)*		*Nephrotoxicity n(%)*		*ACR n(%)*		*Sepsis n(%)*	

PPARA48 rs4823613	(+)	(-)	(+)	(-)	(+)	(-)	(+)	(-)	(+)	(-)	(+)	(-)
AA(n=23)	9(11.5)	14(17.9)	6(7.6)	17(21.7)	5(6.4)	18(23.0)	2(2.5)	21(26.9)	3(3.8)	20(25.6)	4(5.1)	19(24.3)
GA(n=50)	16(20.5)	34(43.5)	9(11.5)	41(52.5)	5(6.4)	45(57.6)	9(11.5)	41(52.5)	7(8.9)	43(55.1)	14(17.9)	36(46.1)
GG(n=5)	2(2.5)	3(3.8)	2(2.5)	3(3.8)	1(1.2)	4(5.1)	0(-)	5(6.4)	3(3.8)	2(2.5)	1(1.2)	4(5.1)
p-value	0.79		0.34		0.32		0.47		0.04*		0.75	

## DISCUSSION

Genotyping of the relevant polymorphisms may predict the response of the drug and decrease the duration for dose titration thereby reducing the probability of adverse events during dose adjustments.^12^-^14^ A genetic polymorphism modifying the structure of the proteins that metabolize drugs may significantly change the pharmacokinetic properties of calcineurin inhibitors eventually causing fluctuations in the time required for dose titration to attain optimum drug levels in the blood.^15^ Polymorphism in CYP3A4 and the interrelated PPARA gene have a crucial role in modifying the clinical response of tacrolimus.^5^,^6^ Our present study analyzed dose-adjusted tacrolimus concentration in transplant patients with CYP3A4 rs35599367, PPARA rs4253728 and PPARA rs4823613 polymorphism. To the best of our knowledge, the present study is the first to report the association of CYP3A4 and PPAR polymorphism with CDR and the adverse effects of tacrolimus in Pakistani liver transplant patients.

The lower number of female transplant recipients in our study conforms to the model for end-stage-liver-disease score (MELD) limitations that prioritize male gender candidacy for liver transplant procedures.^16^

Our results show that the CYP3A4*22 rs35599367 homozygous GG genotype reported significantly lower CDR in comparison to the CYP3A4*22 rs35599367 T-allele carriers during the first two weeks in the post-transplantation period. Our results are in agreement with the findings that have reported that rs35599367 C>T polymorphism alters the structure of CYP enzymes leading to less activity thereby resulting in the elevated CDR of many drugs.^7^ Our results are confirmed by studies that have reported significantly reduced tacrolimus dosing requirement in transplant recipients with rs35599367 C>T polymorphism.^17^,^18^

The function of the CYP3A4 proteins can also be affected by factors outside the CYP3A4 gene that can ultimately modify the rate and extent of metabolism of drugs; two such examples are the polymorphisms at rs4823613 and rs4253728 in the peroxisome proliferator-activated receptor-α (PPARA) gene.^8^,^19^

According to our findings, the GA and GG genotype at PPARA rs4253728 genotype consistently showed higher tacrolimus CDR than AA genotype during the first four weeks of the post-transplantation period though this value was not statistically significant. According to our findings, tacrolimus trough levels for GG genotype at PPARA rs4823613 were consistently low during weeks 1-4, although these values were also not found to be statistically significant. Our findings conform to similar results in renal transplant recipients in the white Polish population that failed to show significant genotype-based results at PPARA rs4823613 SNPs and rs4253728 throughout the first three months of immune supression.^20^

According to our results, a significant association was found between the absence of psychosis, seizures and sepsis with CYP3A4*22 rs35599367 homozygous CC genotype and these findings could be due to the significantly reduced tacrolimus trough levels in homozygous CC genotype leading to reduced incidence of adverse effects.^21^

As per our findings, a significantly higher number of individuals with CYP3A4 rs35599367 CT genotype developed renal toxicity which might be a result of the significantly elevated tacrolimus trough levels in these recipients. Our results are confirmed by literature that has reported renal toxicity as a significant adverse effect due to high tacrolimus trough levels.^21^-^23^

Our results showed that any adverse effect was not significantly associated with PPARA rs4253728; these findings are in agreement with the results that failed to show a significant association of polymorphism at rs4253728 with neurotoxicity and nephrotoxicity.^24^ The absence of ACR was found to be significantly associated with GG genotype at PPARA rs4253728. A probable reason for this significant association may be the consistent fluctuations in tacrolimus CDR observed in the AA and GA and genotype; CDR of individuals with GG genotype did not oscillate as much as the former two genotypes; stable levels of tacrolimus may shorten the time required for dose titration thereby reducing the probability of developing ACR and other adverse effects.^21^ PPARA rs4823613 polymorphism also showed similar findings and none of the genotypes showed significant association with adverse effects. The absence of ACR was significantly associated with GA genotype which could also due to stable blood levels of tacrolimus resulting in less time for dose titration thereby reducing the probability of adverse events.^21^

## CONCLUSION

A significant association exists between CYP3A4 rs35599367 polymorphism with neurotoxicity and nephrotoxicity and between PPARA rs4253728 and rs4823613 polymorphism with sepsis in Pakistani liver transplant recipients taking tacrolimus. CYP3A4*22 rs35599367 polymorphism may lead to fluctuations in tacrolimus dose requirement during dose titration. We propose that identifying polymorphism in CYP3A4*22 rs35599367 might reduce the time required during tacrolimus dose titration and may predict adverse drug events.

### Authors Contribution:

**FA:** Conceived and designed study; collected and analyzed data; drafted manuscript.

**MK, AS:** Designed study; interpreted data and drafted manuscript, reviewed final draft.

**ABH:** Designed study; analyzed data; reviewed final draft.

All authors are responsible and accountable for the accuracy and integrity of the work.
